# The experience of international nursing students studying for a PhD in the U.K: A qualitative study

**DOI:** 10.1186/1472-6955-10-11

**Published:** 2011-06-13

**Authors:** Catrin Evans, Keith Stevenson

**Affiliations:** 1 School of Nursing, Midwifery and Physiotherapy, University of Nottingham, B Floor, Queens Medical Centre, Nottingham, NG7 2UH, UK; 2 School of Health, Glasgow Caledonian University, Cowcaddens Road, Glasgow, G4 0BA, Scotland, UK

## Abstract

**Background:**

Educating nurses to doctoral level is an important means of developing nursing capacity globally. There is an international shortage of doctoral nursing programmes, hence many nurses seek their doctorates overseas. The UK is a key provider of doctoral education for international nursing students, however, very little is known about international doctoral nursing students' learning experiences during their doctoral study. This paper reports on a national study that sought to investigate the learning expectations and experiences of overseas doctoral nursing students in the UK.

**Methods:**

Semi-structured qualitative interviews were conducted in 2008/09 with 17 international doctoral nursing students representing 9 different countries from 6 different UK universities. Data were analysed thematically. All 17 interviewees were enrolled on 'traditional' 3 year PhD programmes and the majority (15/17) planned to work in higher education institutions back in their home country upon graduation.

**Results:**

Studying for a UK PhD involved a number of significant transitions, including adjusting to a new country/culture, to new pedagogical approaches and, in some cases, to learning in a second language. Many students had expected a more structured programme of study, with a stronger emphasis on professional nursing issues as well as research - akin to the professional doctorate. Students did not always feel well integrated into their department's wider research environment, and wanted more opportunities to network with their UK peers. A good supervision relationship was perceived as the most critical element of support in a doctoral programme, but good relationships were sometimes difficult to attain due to differences in student/supervisor expectations and in approaches to supervision. The PhD was perceived as a difficult and stressful journey, but those nearing the end reflected positively on it as a life changing experience in which they had developed key professional and personal skills.

**Conclusions:**

Doctoral programmes need to ensure that structures are in place to support international students at different stages of their doctoral journey, and to support greater local-international student networking. Further research is needed to investigate good supervision practice and the suitability of the PhD vis a vis other doctoral models (e.g. the professional doctorate) for international nursing students.

## Background

Globally, a key strategy for the attainment of the millennium development goals and health system improvement is the development of nursing capacity. There is increasing investment by governments in nurses to attain doctoral degrees with the expectation that these graduates will then be able to lead the advancement of nursing in their respective countries [1]. There is a global shortage of doctorally qualified nurse educators and of doctoral nursing programmes however, which means that many international nurses have to go overseas to obtain their doctorates [2]. The USA, Canada, the UK and Australia currently comprise the major destination countries [3,4].

Study abroad involves a large personal, social and financial investment on the part of individuals, their families and employers. High hopes are being pinned on the shoulders of doctorally prepared nurses [5], yet relatively little is known about the learning experiences of these students during their studies overseas [6]. What is it like to try and achieve the highest possible academic award in an unfamiliar context? Is the PhD programme what students expected? What are the challenges? How useful and relevant is the doctorate perceived to be? How can supervisors and other doctoral educators best support this student group? This paper describes a study that set out to explore the international doctoral journey in the UK. In doing so, it adds to a slowly growing body of evidence exploring the nature, quality and appropriateness of different doctoral nursing programmes in a globalised context [7-10]

### The UK Doctoral Nursing Context

Currently, UK nursing doctorates consist of two main programme routes - the professional doctorate and the 'traditional' PhD. The former is offered in many universities and, although there are some local variations, it usually includes an initial structured programme of cohort-based taught courses followed by a period of autonomous research leading to a dissertation. The professional doctorate is aimed primarily at practitioners who wish to apply a more evidence based approach to their work. Hence, it is orientated towards applied clinically relevant research and its taught elements usually include research training, clinical leadership and practice development. The practice-based doctorate that is gaining popularity in the USA as the entry level for advanced nursing practice is not yet offered in the UK, but one such programme has recently started in Ireland [11].

Students undertaking a PhD are usually also required to undertake formal research training in the form of taught courses, however, the training programme can be more flexible as the PhD has traditionally been envisaged as a more individualised programme of self-directed study relating to the planning and completion of a piece of original research. The majority of UK doctoral programmes also include substantial provision of transferable skills training to enhance graduate employability [12,13]. These are primarily delivered through inter-disciplinary 'Graduate Schools' and cover the following areas: research skills and techniques, research environment, research management, personal effectiveness, communication skills, team-working and networking skills, and career management [14].

In the UK, both the PhD and the professional doctorate take approximately 3-4 years full-time or 6-8 years part-time. In most UK universities, students are supervised by a team of 2 supervisors who meet with their student to discuss progress at least once a month.

According to the UK Higher Education Statistics Agency (HESA), in 2007/08, approximately 15% of UK doctoral nursing students were international (124 EU/non-EU students, 692 UK resident students) [15]. The majority of UK-resident doctoral nursing candidates are on part-time programmes however, so international students constitute the majority of full time doctoral students in many nursing departments [15].

### The International Doctoral Nursing Student Experience

A recent literature review carried out by the authors synthesised a wide body of evidence relating to overseas doctoral students [6]. The review highlighted the transitional nature of the international doctoral student experience, suggesting that students face a range of cultural, pedagogic, linguistic and social adjustments [16,17].

A particular issue that many studies identified was the challenge of adapting to student-centred, self-directed learning practices common to western countries (as opposed to more didactic approaches that are often found in other parts of the world) [18,19]. The review also points out that the nature of the student experience is strongly influenced by their disciplinary affiliations and traditions and that this is turn is related to the kind of supervisory relationships that can develop [20,21]. In faculties of science for example, doctorates tend to be undertaken as part of a team of researchers whereby students have opportunities to gain support from peers and supervisors on a regular basis [22]**. **In social science doctorates however, the study context may be much more isolated making the process of adjustment more difficult [23]. Very little is known about how the particular disciplinary context of nursing affects the international doctoral student learning experience.

To our knowledge there have only been 3 nursing-related studies published on the international doctoral student experience One (published in 1995) was a retrospective phenomenological interview study conducted amongst 23 Taiwanese nursing graduates of US educational programmes, 4 of whom had completed doctorates [24]. Although they were now proud of their academic success, the respondents described their study abroad period as one of enormous stress, hard work and loneliness, characterized by little interaction with US nursing students. Another study (published in 2007) from one institution in the UK presented data based on a survey completed by 5 international doctoral students and 11 research supervisors [25]. Both staff and students saw great value in international education. However both groups identified the need for greater support to facilitate adjustment in a number of areas, including: understanding the PhD process, studying in a second language, working within a different academic culture, managing the supervision relationship, and finding a sense of community. Recommendations included staff training and the development of additional in-puts to support students. A larger scale study published in 2002 in the USA reported on a national survey exploring the international student experience completed by 24 different Schools of Nursing [26]. Challenges for students identified by the faculty included language and communication, development of critical thinking skills, inadequate financial support, loneliness and isolation. The faculty reported enjoying working with this student group but felt unable to give sufficient time to meet their needs, suggesting that more help from the wider university systems was required. This survey was followed up with one focus group with 5 international doctoral students at one US university. The students identified a range of similar challenges: lack of familiarity with US health care system; lack of familiarity with US teaching practices; lack of opportunity to participate in faculty research; communication problems and stress from trying to cope with a heavy workload. Although rather small scale, all three studies indicate that studying overseas can be stressful, although more in-depth data on the nature of specific challenges or ways in which students might be supported through these was rather limited. These studies are now rather out of date. Doctoral programmes and infrastructure are changing rapidly, particularly in response to enhanced global student mobility. More in-depth and contemporary research is required to build up a picture of the current situation.

### Research Aim

The study aimed to explore the international doctoral student journey; specifically, to investigate the learning experiences of international doctoral nursing students at different points in their PhD journey and to identify best practice in supporting effective learning in this student group.

## Methods

### Research Design and Methodology

The study adopted a descriptive qualitative approach. A descriptive approach is considered appropriate when the features of particular phenomenon are not yet well understood. The study employed semi-structured interviews to give primacy to students' own perspectives and to the uniqueness of their individual experience [27,28]. A cross-sectional design was adopted whereby each participant was interviewed once at a particular point in their doctoral journey. The research adopted a constructivist methodological approach [29], recognising that students' accounts of their learning experiences were socially constructed, reflecting their own unique lives and backgrounds and reinterpreted for a researcher during the interview process.

### Recruitment and Data Collection

Data was collected in 2008/09. Information letters were sent to all Schools of Nursing in the UK that were identified as running a doctoral programme (n = 44). The letters requested permission to access their doctoral student groups. Of the initial 44 Schools, only 21 reported having any international doctoral students. Of these, 3 declined to participate and 5 did not respond to repeated follow ups. The Heads/Deans of the remaining 13 Schools were then requested to forward one initial and one follow up research information email to their doctoral students and to put up research recruitment posters in student offices. Seventeen students from 6 different universities across the UK volunteered to be interviewed. It is not clear why students from only 6 universities responded - we did not attempt to ascertain whether all 13 participating Schools had indeed followed through with the requests to advertise the study. The interviews were arranged and conducted by a research assistant at a location of the students' choice and lasted between 1-3 hours. The research assistant was an international doctoral graduate (although not a nurse), whereas the other researchers were UK academic staff who led doctoral programmes. The latter were not directly involved in interviewing since it was felt that students would feel more able to open up freely about their experiences to a researcher who was not a UK academic member of staff. In order to enhance reliability or dependability [30], all the interviews were listened to by the principal investigator and a de-briefing meeting was held after each one to monitor quality, to discuss any issues that arose and to ensure consistency within the interviewing process [31].

Ethical approval for the study was granted by the University of Nottingham Faculty of Medicine and Health Sciences Ethics Committee. All students who agreed to participate signed a consent form and were provided with a book voucher as a token of appreciation after completing the interview.

### Data Analysis

All the interviews were digitally recorded, transcribed and checked for accuracy. Field notes were taken immediately following each interview to provide context and individual case summaries were developed to facilitate analysis of each individual's doctoral experience.

The interview guide asked students to explain why they had chosen the UK and their particular university and type of doctoral programme. They were then asked to talk freely about their experiences in the UK and with the doctorate so far.

Analysis was an on-going iterative process. Trustworthiness of the study was enhanced by a variety of analytical strategies, as suggested by Lincoln & Guba [30]. To maximise credibility, the data was initially analysed jointly by the research team with the aid of NVIVO. Each researcher read the transcripts a number of times and assigned a code to significant units of the text. There was significant inter-coder agreement, but inevitably, the researchers' different experiences and standpoints influenced the coding process to some extent. We engaged in numerous reflexive exercises to clarify our own positions and preconceptions [32]. Any variations in coding were extensively discussed and the transcripts repeatedly revisited until a jointly agreed coding framework was developed which continued to be revised and refined over time. The codes were then clustered into a range of emerging themes and sub-themes which were then grouped into 3 major categories - see table 1[33,34]. Careful attention was paid to atypical cases and specific examples of these are noted in the findings [35].

**Table 1:** Analytical framework

Categories	Themes
1. A Journey of Transitions: Adjusting to Doctoral Study in the UK	• Expectations and reality
	• Anxiety and challenge: adjusting to UK academic practices
	• Learning in another language

2. A Journey of Relationships: Finding Support for Doctoral Study	• Negotiating the complexities of supervision
	• Peer support
	• Institutional support

3. A Journey of Challenge and a Journey of Growth	• An emotional journey
	• Transformation

### The Participants

The 17 participants represented approximately 14% of the possible international doctoral nursing student population (n = 124) [15]. Table 2 summarises the broad socio-demographic characteristics of the research participants. Some details have been kept deliberately non-specific (e.g. nationality) in order to protect the anonymity of the participants. The number of students interviewed per university ranged from 1 to 5. The students came from 9 different countries. The majority of students were mid-career, both in terms of age and position. Many students (n = 9) were already working in higher education (e.g. as clinical instructors or junior lecturers) and needed a PhD in order to progress further. The majority of these were involved in pre-registration nurse training (rather than specialist post-registration or MSc level education). Likewise, some participants (n = 7) felt that they had reached the end of their clinical career ladders and that their only way forward was a lateral move into higher education which necessitated a PhD. Three of these were senior nursing administrators in hospitals, one was a palliative care specialist, one was a diabetes specialist and two had backgrounds in critical care nursing. Most participants (n = 15) saw their future careers in higher education. The majority (n = 13) were supported via government scholarships and all of those who were married had been able to bring their families. Six participants were male. Four of these were in the UK with their families. The other two were unmarried. Out of the eleven female participants, six were in the UK with their husbands/children; five were unmarried and had come alone. All of the participants were undertaking traditional 3 year PhD programmes involving the student carrying out a piece of original empirical research, presented in a thesis written in English and examined by viva conducted in English.

**Table 2:** Characteristics of the sample, n = 17

Nationality	Students were from 9 different countries (2 EU, 15 non-EU). The main regions were EU (n = 2), Middle East (n = 8), East Asia (n = 4), South Asia (n = 2) and sub-Saharan Africa (n = 1).
Gender	Male: n = 6, Female: n = 11

Year of Study	Ranged from 1-5 years as follows: year 1 (n = 3), year 2 (n = 4), year 3 (n = 7), year 4 (n = 2), year 5 (n = 1). The majority (n = 10) were in the final stages (years 3-5)

Source of Funding	Most had government scholarship (n = 13); the rest were self-funded.

Age	The majority (n-10) were <32 years; the rest (n = 7) were aged between 33-49 years

Social situation	The majority (n = 10) were in the UK with family; the rest were on their own

Pre-doctoral employment situation	Higher education (n = 9), senior clinical practice (n = 7), policy work (n = 1)

## Results

### A Journey of Transitions: Adjusting to Doctoral Study in the UK

For many of the study participants, undertaking a PhD in the UK involved a number of significant transitions requiring a process of adaptation, learning and adjustment.

The first of these was the need for some students to adjust their expectations of the PhD programme structure and content to the reality that they encountered. Many participants had expected a highly structured academic programme with a strong emphasis on course work as well as research (more akin to the professional doctorate). They expressed great surprise that they were expected to develop their own programme of work according to their own learning needs:

When I came and start doing my PhD I never thought it would become purely dependent on the student, and the supervisor just will give you headings, or guidelines, I thought it was like, just a total programme......... I didn't think it would be pure research, just by learning by yourself (S.16)

Likewise, many students (n = 13) noted that they had expected a greater focus on professional nursing issues within their programme and were surprised at the almost exclusive emphasis on research. Some students had expected to undertake clinically-oriented specialist courses alongside clinical practice, as was their experience from their own countries:

Clinical theory it's more important, it's like we already told our Dean of the department here - we need it because we come here and you can find nothing. There is always a gap between your theory and the practice. And why we do PhD but we don't have clinical experience? It's so tricky you know; you know it's a problem but you still not operate. In my country I do my Master's, I still need to do the placement in hospice ward, I learn it, and my supervisor had to go to the hospice ward to supervise me and discuss meeting about my case study. Yes, I think it's important (S.8).

Others were not specifically seeking further specialist courses but had expected the PhD programme to include more input on understanding the UK nursing situation. Only two mentioned having spent time visiting clinical settings during their time in the UK. A number of students commented that this lack of engagement with UK nursing had hindered their ability to reflect critically upon nursing in their own countries or to act as agents of change upon their return:

This is the problem we all have now. If we go back to our country someone will say 'what is UK healthcare system'? We don't know, because we don't have a chance to go into the field, we didn't have a chance to observe. We just do our research, in our field only, in office, face the computer. How can we know nursing here? It would be very useful because we are looking for international comparisons... especially for me as a change agent. My government to send me here to the UK, so it's expected when I go back to my home country I will make some changes and so would like to learn more about what is going on in the UK here so I can transfer that knowledge to my home country. (S.14)

When asked why they had chosen the PhD rather than the professional doctorate, the majority of participants said that they had never heard of the professional doctorate programme and did not know anything about it. Those that had heard of it thought it required a compulsory clinical practice component (which is not the case in the UK model of the professional doctorate) and therefore excluded themselves on the grounds of not having UK nurse registration. In addition, most participants felt that it would not be recognised in their own countries.

A second transition for students was the need to adapt to the self-directed autonomous nature of learning at doctoral level. Many participants found this extremely difficult, especially in the first year, when they were trying to find a focus and identify their research questions. Some students described how the need to define and take the lead on their own projects had created deep anxiety and a desire for more guidance:

I think I spent quite a lot of time trying to understand what I need to do. The project is specific just for me, so I cannot ask anything from my classmates. Then, I cannot make this plan because I want my supervisor to tell me it is right or wrong, but every time my supervisor asked me what are you going to do next? What's your plan? And I just can't think, oh I don't know, I thought you would tell me, you know, like that. (S17)

Others however noted that, although deeply challenging, independent learning had been a liberating and exciting endeavour:

There's a certain freedom in the PhD.............but you always need to motivate yourself and mobilise yourself in different ways in order to get the results. This is sometimes exciting, sometimes it's disappointing, frustrating, but in total I would say it's an enviable experience for me. (S.13)

A third transition was the need to understand and adjust to the expectation of originality and criticality in doctoral level work. Many students noted that their educational backgrounds had trained them to describe and replicate knowledge rather than to create it. They commented that their doctoral work had initially reflected this descriptive approach and that developing criticality was a long and slow process. Students frequently associated personal development in this area with particular supervisory practices (see below) such giving detailed feedback and discussing specific texts with students. Several students noted that, although challenging, they had come to enjoy 'finding their own voice', noting that their growing ability to articulate their own ideas and to contribute to academic debates was empowering:

In my country, for exams we had to memorise. In assignments, I used to prepare them by taking paragraphs from different books, this one here and that one there - a collection of paragraphs is a Masters assignment. But now, finally, I know how to create - I can find out evidence - I can create an original draft from my original ideas, - not plagiarised, it's my own. I can accept others' argument, but at the same time now I can argue my own argument, that is a skill I learnt from learning here for two year. (S3)

A fourth transition that affected 16 of the sample was the need to learn and write in a second language. These students strongly emphasised the enormous challenge of studying in English. For them, social interaction and academic study initially demanded a huge effort which created stress and anxiety because of the extra time and energy that every task required. In addition to learning to study in a different language, students also commented that they needed to adapt to different expectations of academic writing, particularly conventions of how to structure longer pieces of work and how to develop a critical academic argument. For many, this had also been a significant challenge:

I think the cultural difference, the way we express ideas - the logic - is different. I think English is very straightforward, you tell the reader what's the purpose, or what's the content, to the paper, and then like a free structure - a,b,c. But in Chinese the logical is different, we just tell you maybe the background and then the story and the end will be a circle to work out whole story and then you work with the end. (S10)

Most of the participants felt that English classes provided by their universities were not very helpful in meeting their language needs. These were considered too basic and too generic. Rather, one to one feedback or discipline-specific language training would have been preferred.

For many students, the transitions described above were most pronounced at the beginning of their doctoral programme study (the first year was often mentioned as a particularly difficult time). In spite of the challenges of adjusting to doctoral study, several students described how their sense of academic capability had grown over time:

If I compare with the first year I felt myself lost when I start, because I wasn't able to plan to exactly what I'm going to do, or to put a timetable for myself, but I think myself now more control over my study, I'm able to do what I'm planning to. I can see the difference, I feel like I have more skills now. (S.4)

### A Journey of Relationships: Finding Support for Doctoral Study

Given the range of adjustments that needed to be made to UK doctoral study, many students described a strong need for support during their doctoral journey. This need for support was identified in the context of three sets of relationships: through supervisors, through interactions with the department/institution and through relationships with other students and wider social networks.

The supervision relationship revealed itself as by far the most significant element of the students' learning experiences. Expectations of the supervision relationship were strongly influenced by the participants' past educational experiences. For example, many participants described their previous student-teacher relationships as having been rather hierarchical whereby students were expected to demonstrate extreme respect and where students were not encouraged to ask questions or to voice their own opinions. This had an impact on the way in which participants engaged with their UK supervisors with many students describing an initial uncertainty in how to approach the supervisory relationship:

I don't know, it's maybe for our culture, we don't talk out, I don't know if it's good or not, because we are afraid to confront teacher, yes. And because I'm afraid that she think that I'm kind of like challenging her or something, but maybe they don't think that way, but we, I think she will kind of like feel differently with me. (S.1)

Likewise, many participants noted that in their own countries, supervisors would be expected to actively lead a project by telling students what to do. For these students, the British expectation of student-led project management was a real challenge:

In my country, if I was doing a Masters or PhD your supervisor would tell you what to do, but here it's different, so I'm kind of like waiting for my supervisor to tell me what to do, but my supervisor is waiting for me to tell them what I'm going to do. (S.8)

In other cases however, the participants favourably contrasted UK supervisors with their home experience and appreciated the time and attention given as well as the more egalitarian and collegiate ethos:

In my country supervisors really don't take time to meet with you like the way we have here. Here, they're very concerned about your welfare, about how you're getting on. (S.16)

In discussing supervision experiences, participants were very clear about what constituted good or poor quality supervision practice - as summarised in table 3. Interestingly, although students clearly valued particular supervisory characteristics such as being knowledgeable or accessible, the most highly valued supervisory attribute was the demonstration of a personalised student-centred approach. This was seen as foundational to the development of trust in the supervisor:

**Table 3:** Student defined characteristics of 'good' and 'poor' quality supervision

Good quality supervision	Poor quality supervision
• Takes a personal interest in the student and in the country and culture	• Too busy
• Engenders a sense of trust and confidence in the student	• Does not seem interested in the project
• Understands the particular challenges for international students	• Task focused, does not take a personal interest - too 'professional'
• Is accessible (e.g. answers emails, is willing to have the odd one-off meeting when needed)	• Student is unsure if they can trust them to guide their project
• Reads work and provides detailed and specific feedback	• Provides inconsistent advice
• Provides a way forward when stuck - suggests new avenues of inquiry	• Inaccessible (e.g. does not reply to emails)
• Provides clear guidance	• Does not read work
• Suggests reading material	• Feedback is too general
• Encourages and welcomes debate	• Throws back questions to the student rather than suggesting possible new directions
• Is an expert in the subject area	• Gives criticism in an insensitive or destructive way
• Challenges the student	• Is not an expert in the subject area
• Acts as a gate-keeper, helping student to identify and take opportunities to build networks and develop skills (e.g. by encouraging the student to present at a conference or to contact an eminent researcher in the field)	

They were friendly, if I want to speak to them for my research or ask a question, first they ask me about myself, about my family and everything, how I'm living, what I'm doing, if everything is OK - this make me believe in my supervisors. When it was critical for me, when I had blocks and couldn't move forward, I trusted their suggestions. The trust is the first important thing and I had that experience in fact, and it helped me to progress with them (S13)

The personalised approach was contrasted unfavourably with supervisors who adopted a task-oriented 'professional' approach or with supervisors who were inconsistent in the way they dealt with students. There was a strong sense that students wanted their supervisors to understand them as people as well as to care about their projects:

I didn't have any interest from my supervisors in terms of sort of emotional support, they were more concerned about research and research only, but not 'is she feeling ok, is she feeling settled'? There are loads and loads of issues, and I think that I wasn't given that much attention from that particular side. You know when there's not that much interest in the student or in their work, you know, how to explain it, the student cannot trust that supervisor. (S.3)

One EU student had a different point of view, expressing satisfaction with purely project focused 'professional' interactions. This student was already highly skilled in project management however and was a senior figure in his field. Unlike many other international students, he was perhaps less reliant on his supervisors to maintain his confidence and to reduce anxiety.

Several participants who were in years 3-5 noted that the supervisory relationship changed over time commenting that there was a gradual development of greater control of their own progress:

There are times when you feel you are very vulnerable with your supervisor. I think for all first year and maybe second year students, they feel they are more led by their supervisors rather than they are in control, but once you sort of gain that sort of confidence and experience and you know your topic which I think you will know your topic more than your supervisors, in that sense you will be able to lead the way. (S.15)

The student experience of finding support from their departments or from social relationships in addition to their supervisors was rather variable. For example, two students felt very isolated because they were the only international students in their departments (and part of a small group of PhD students more generally). Others noted that they had developed good links with other international doctoral students (primarily due to physical proximity when sharing an office) but all commented that it was difficult to get to know UK PhD nursing students. Many commented that UK PhD nursing students are usually part-time and are thus rarely in the department. Others mentioned that they felt shy about approaching UK students and several expressed a feeling that the UK students seemed too busy or not particularly interested in their international peers.

You know, most of the British students study part-time, so we don't see them all the time. I had in fact several attempts to be involved with them, but I think that it is like I am shy. When we meet them in some classes they are good, they are helpful but they are busy on their own or maybe I'm not friendly enough to them so that I can continue with them (S.8)

Some Schools organised regular PhD student and/or staff research seminars and students placed great value upon these as useful arenas for relationship building:

The seminars helped me meet other PhD students. When you meet and chat with other people you will feel that it's not only you in this world, you're not on your own, because sometimes you feel 'I'm the only one who's doing a PhD, no one else is doing it'. So they will tell you about their problems or their difficulties, and they're living in misery now because they couldn't do so and so. So it's also social event let's say, yes. We exchange telephone numbers and email - it becomes a network (S.11)

The ability of students to develop relationships with other staff in their School was also variable. In some cases students felt that academic staff did not always take the time to get to know them and did not recognise the skills and experience that they brought with them:

We didn't see much initiation or willingness from some of the staff to know the students. When people will ask you 'oh where are you from, you are from [country name]'. OK - fine, then they just assume that you don't know nothing. I think they need to learn about, or to know about the background of international students (S.9)

Where good relationships developed however, students had sometimes found opportunities to work as research or teaching assistants and felt that they had gained valuable transferable skills, as well as building wider social networks.

A large number of students commented that a whole-School approach to international students was needed, in which all staff, including administrative and cleaning staff displayed a positive approach and where systems were in place to meet student support needs:

I feel that helping the international students and being successful with the international students is not one person's work, it is team work and imagine that one member of the team is not professional and not helpful, the whole process will fall down, it is like a chain and part of this chain is the administrative staff, the cleaners, the technicians, it's not the academics only and that is a very, very important part (S.5)

Outside the department, some students had been able to develop enjoyable and supportive social links (although these were primarily within their own religious or ethnic communities or with other international students). A number of participants mentioned that social events organised by the university had been an important factor in developing friendships and having an occasional break.

Those students with stronger social networks (particularly those who had come with their families) seemed more positive about their overall PhD experience.

### A Journey of Challenge and a Journey of Growth

The majority of participants indicated that the PhD felt like a long, emotionally and academically challenging journey - see table 4. The academic transitions and challenges took place in the context of adjustment to leaving home and learning to live in a new country and culture. Thus, many participants described struggling with loneliness, isolation and the cost of living in the UK:

**Table 4:** Challenges and achievements during the PhD journey

Challenges	Achievements
• Stress	• Increased confidence
• Anxiety	• Opened eyes
• Self-doubt	• New ways of thinking
• Homesickness	• Increased self-esteem
• Suffering	• Independence
• Financial difficulties	• New friends
• Family pressure	• New knowledge and skills
	• Transformation of self

Sometimes you feel you are an alien here. How to say? The world feels upside down. I am away from my home country, from my family. I feel very lonely because I come from an extended family with many people living with me, so I found staying alone in this country is very difficult. Here you are totally in charge of doing everything by your own, this puts a lot of stress on you (S.9)

Most participants carried the weight of high expectations from family, colleagues and sponsors on their shoulders which, in some cases, had clearly led to chronic anxiety about whether they would succeed. In some cases, this prevented students from being able to engage in social aspects of life in the UK and prevented them from being able to enjoy their PhD experience:

I can say, I am inventing a new knowledge. So that's internal satisfaction. When my supervisors say 'good' I feel a reward and a relief. But externally there is, you know, there is nothing - just worry. At home everyone is waiting for me. So then I am thinking - will I do it? can I finish this? I mean I'm not really enjoying you know the PhD life. Sometimes I try to enjoy in the UK, but I feel I have to finish, only then can I enjoy (S.14)

As noted above, students who had come with their families had wider social networks and some spoke positively of the support they had received from their spouse and children. On the other hand, both male and female married participants commented that their families required precious time, attention and financial resources, noting that it was sometimes very stressful trying to juggle conflicting demands. However, amongst the 7 unmarried participants, the 5 females in particular expressed considerable loneliness. This was especially marked amongst those from the Middle East who were used to living in extended family situations in a context where everyday activities of life were often undertaken collectively and who now found themselves having to manage everything alone:

Living on my own, and having to manage everything by my own, it was completely different from how I used to live back home. I had, you know - there are always your brothers, your sisters around you, your mum, your father supporting you, but here you have to do everything on your own (S.8)

Almost all the participants were used to being high achievers and, in some cases, had enjoyed social prestige from being in highly respected positions in society (e.g. a lecturer). The interviews showed that, for a significant number of students, this self identity was challenged as they struggled to adjust to UK academic practice but also to the social status of being a student again:

My supervisors accused me of poor work after I spend so long on the writing. You know this really hurts your self esteem, yeah. When I think - before - you know - I had my own office. People come and take my bags, bring me tea but now, you know, I'm here as a small stuff, suddenly everything change. This hurts our confidence (S.12)

On a more positive note, students at a more advanced stage in their studies talked at length about how much they had developed throughout the course of the PhD. Much of their discussion focused on the personal development that stemmed from adaptation to a new country as well as academic development as a result of their studies. Many noted that their confidence and their ability to manage their life and work independently had grown tremendously. This appeared to be particularly pronounced for the female participants, especially those from the Middle East:

Well it's been a massive experience for me, massive. I mean personally, emotionally, intellectually, mentally, I think I have grown up a lot. My confidence has increased most. Before, when I worked as a nurse I didn't want to talk to others, but now I'm more confident, I can talk with people in different way, I can express myself in a different way. I think part of it is to do with the topic and the project itself and the other part is about living in the UK. Being on my own in the UK all these years, I think that was the biggest challenge and that taught me a lot. I think I will definitely apply the skills that I have gained from living here, whether it is in building my confidence, whether it is interpersonal skills, communication skills, I think I have learnt a lot. Now I'm totally independent person. (S.15)

Interestingly, although adjusting to 'independent study' was one of the biggest challenges that students said they faced, many also described tremendous satisfaction at having developed independent research and problem solving skills. Some participants described how their whole outlook had changed as a result of their experiences in the UK, noting a general development in their intellectual maturity:

The way how we analyse things after you have completed your PhD is different than when you started. You see different way of analysing, you see different perspectives, and you learn how the others think about the same events. You just have one way of thinking and the others have others, so we can share the perspectives. I learn how to be a high professional person, and to respect others obedience, I learnt how to present myself at my study, I learnt also how to be open and accept the others opinions, accept the criticism. I learn too many things, I feel like I really become a different person than when I just came here. (S.16)

In sum, for many participants, the PhD was experienced as a stressful but transformative journey.

## Discussion

This study has identified five factors associated with doctoral study in the UK that affected the nature of the learning experience for overseas doctoral nursing students.

First, there was a gap between students' expectations of PhD study and the reality that they encountered. Whilst PhD programmes will inevitably vary from one institution to another, many students expected their PhD to include a stronger professional nursing focus in addition to the research emphasis - more akin to the taught professional doctorate model. Several students expressed regret that their PhD had not afforded them with greater opportunities to learn more about UK health care practice, and, therefore, to equip them with a broader vision of global healthcare. Although students clearly wanted greater engagement with professional nursing issues, they all felt that any doctorate other than a PhD would not have been recognized in their own countries. This finding relates to a wider debate on the growth, recognition and international relevance of the professional doctorate in nursing and suggests that there may be a need for greater promotion of professional doctorate programmes to the international nursing community [10]. In the short term however, this finding also indicates that existing PhD programmes may need to create a formal system by which to facilitate clinical insight visits and to provide courses or seminars that enable students to understand and critically compare UK healthcare practice from an international perspective. Likewise, research supervisors could be trained to help students to make relevant clinical connections.

Second, our findings show that many of the participants initially struggled to understand and develop key doctoral level skills within the context of UK academic practice (specifically criticality, self-directed learning and English language/writing) - although those students with previous experience of UK study seemed to have an easier transition into doctoral academic practice. As the interviews focused only on the nature of the student experience (rather than evaluating the content and structure of each institution's doctoral programme), it is not possible to draw any firm conclusions regarding which aspects of a doctoral programme can best support the development of these skills. This is an area that requires further research [36,37].

It is important to point out here that the move to doctoral level being and thinking is a transition that all doctoral students need to take, not just international students [36,37]. By focusing upon international students, we do not wish to artificially problematise this group [38]. There is still much to be learned about how institutions and supervisors can best support the development of doctoral students into confident autonomous researchers, and how the needs of specific student groups can be addressed within this process [36,37]. A number of authors have commented that the current model of doctoral education can create hurdles for many types of 'non-traditional ' student (e.g. older students or part-time students), and it may be that many of the challenges raised by the international students in this study are shared by other student groups [39-42]. Indeed, we suggest that several issues raised in this study may well apply to other groups of doctoral nursing students. For example, studies on the professional doctorate suggest that UK nursing students also face challenges in making the transition to independent research and that their adjustment is hampered by their part-time status which acts as a barrier to engagement with peer networks and an institution's wider research environment [43-45].

Third, many participants wanted more structure within their PhD programme (expressed as wanting more taught input and more guidance). The majority of UK Nursing PhD programmes do in fact offer extensive research methods courses and encourage students to undertake these. These courses are taken on the basis of individual need however rather than as part of pre-defined structured programme so many of the study participants still expressed a sense of uncertainty and lack of direction regarding their progress and plan of work. This uncertainty was most acute during the first year of doctoral study - a finding that has been reported in other studies [46,47]. Whilst this suggests a need for more support, there is also a growing recognition that going through a degree of anxiety/uncertainty is an almost inevitable part of developing a doctoral identity as research students slowly come to find their own epistemological positions, to define their own research questions and to create their own voice [48]. The participants' accounts of self-transformation suggest that they did indeed experience their doctoral study as life changing. Those in the later stages clearly valued their new research-related knowledge and skills but also appreciated more generic changes in their own personalities (e.g. becoming more confident and independent) and in the way in which they approached the world (e.g. becoming more open minded, tolerant and more able to solve problems).

Fourth, the research found that the doctorate was an emotionally laden journey and that many students experienced high levels of stress and anxiety during their studies. Whilst some of this stress was associated with the personal, academic and cultural transitions that were demanded, it was also strongly related to the perceived quality of the supervision relationship. The research found that supervisors constitute students' primary sources of support and guidance and play a key role in the development of doctoral level attributes. As in other studies [16,17,19], our findings suggest that the experience of 'good' supervision is predicated upon a personalised relationship of trust and emotional support. Doctoral supervision is still an under-researched and little understood area of pedagogical practice [49,50], and there is a particular need for more work to be done to identify and disseminate models of good practice in supervising international doctoral students [51,52]. Our study also indicates that supervisors may need more training and support to understand and work effectively with this student group [53].

Fifth, the dependence upon supervisors found in this study may also have been related to the fact that only a few students had developed strong links with other elements of a university's research environment. For example, relatively few participants had been able to forge close links with other (UK) doctoral nursing students and relatively few seemed to have become engaged in wider departmental research activities. This finding has also been reported in previous studies of international doctoral nursing students [24-26]. Thus, the PhD seemed to have been experienced as a journey taken in isolation rather than in the context of a supportive peer community [21]. Increasingly, the development of professional networks are considered essential for socialisation into the role of independent researcher [14]. Moreover, the development of international-home student links is important in order to build future communities of practice of nurse researchers. Given the students' initial unfamiliarity with the UK context, the onus appears to be on the School and/or the supervisors to proactively create regular opportunities for networking and relationship building [40]. The research also shows that other staff (including administrative staff) need to be made aware of international student needs and encouraged to make an effort to make them feel welcomed and supported.

### Recommendations for Best Practice in Supporting Learning for International Doctoral Students

Overall, the findings suggest that Schools of Nursing need to create an infra-structure and whole-systems approach to 'scaffold' the learning of international students within their doctoral programmes. Such an approach should aim to support the transition to doctoral level study, to create a sense of confidence in their direction of travel, to support the development of productive supervisory relationships and to support engagement with other social and academic/research networks. A range of possible scaffolding inputs is summarised in table 5. A key element of this approach is supervisor training to ensure that supervisors understand the particular needs of this student group and develop effective strategies for providing feedback and guidance.

**Table 5:** Recommendations for scaffolding the PhD programme

Adjusting to doctoral study	• Clear pre-admission information and pre-admission discussions to clarify expectations.
	• Induction and orientation process, especially in the first year, including language and study skills support
	• Create a more structured 'feel' to the programme
	• Consider developing professional nursing content and clinical links within the programme
Finding support for doctoral Study	• Induction should include orientation to UK supervision practice
	• 'Integrative events' to develop peer networks & better networking with School staff
	• Pro-active whole systems approach
	• Supervisor training but also training for other staff

Personal challenge and Personal growth	• Facilitate access to university sources of support and social events
	• School-level international student advisor

### Limitations

The most significant limitation of this research is its cross-sectional design which provided only a snap-shot of student views at a particular point in time, and in so doing limited the conceptualization of the PhD as a long term learning process. The fact that the sample included students at different stages of the PhD overcame this limitation to some extent, but a longitudinal design would provide opportunities to gain a more in-depth understanding of how the PhD experience unfolds. It is not clear how representative the sample was. Due to self-selection, it is possible that it included students with particularly strong views.

Respondent checking to enhance trustworthiness (particularly credibility) is a contested concept in qualitative research, and this was not undertaken in a formal manner as many of the participants had graduated and returned home by the time the final study report was completed [54]. Nonetheless, a number of seminars about the research were held with international doctoral students based at one UK University and at one national event on teaching international students. All those present strongly endorsed the analytical framework presented here, commenting that it mirrored their own experiences. In addition, the findings are highly consistent with the existing literature in this area, lending some support to their wider transferability [55].

## Conclusions

The further development of doctoral educational practice in nursing is hindered by a very limited evidence base. Quality criteria have been defined [8], but, with some exceptions, there have been very few evaluations of doctoral programmes for nurses [7,56-58]. It is unclear to what extent the issues reported in this study are similar or different to the experience of international doctoral nursing students in other popular 'doctoral provider' countries or to the experience of 'home' doctoral nursing students. Given the strategic importance (and scarcity) of doctorally qualified nurses, more research is needed to ensure a high quality doctoral provision and a satisfactory student experience.

This particular study has provided important insights with regard to the learning experiences of international doctoral nursing students in the UK and has suggested areas where further support and programme development may be beneficial. Further research is needed to investigate the suitability of the PhD *vis a vis *other doctoral models for this student group.

## Competing interests

The authors declare that they have no competing interests.

## Authors' contributions

CE conceived of the study. CE and KS supervised data collection and conducted the analysis. CE drafted the initial manuscript. KS revised parts of the manuscript. Both authors have read and approved the final manuscript.

## Pre-publication history

The pre-publication history for this paper can be accessed here:

http://www.biomedcentral.com/1472-6955/10/11/prepub
